# Prediction of Long-Term Survival after Coronary Artery Bypass with Bilateral Internal Thoracic Artery Grafting: External Validation of A Contemporary Nomogram

**DOI:** 10.3390/jcdd9110375

**Published:** 2022-11-02

**Authors:** Ioannis K. Toumpoulis, Dimitrios A. Kanistras, Christina K. Pappa, Zoi Zagoriti, Constantine E. Anagnostopoulos, Stavros K. Toumpoulis

**Affiliations:** 1 Department of Cardiac Surgery, Mouwasat Hospital, Dammam 32263, Saudi Arabia; 2 Department of Cardiac Surgery, National and Kapodistrian University of Athens, 12462 Athens, Greece; 3 2bull MeDiTherapy P.C., 26222 Patra, Greece; 4 Department of Cardiac Surgery, Mount Sinai Morningside/St. Luke’s Hospital Center, New York, NY 10025, USA

**Keywords:** coronary artery bypass grafting, bilateral internal thoracic artery, risk score, long-term survival

## Abstract

Background: This study aimed to verify the external validation of a contemporary nomogram in predicting long-term survival after an isolated coronary artery bypass with bilateral internal thoracic artery grafting (CABG-BITA). Methods: Consecutive patients who underwent CABG-BITA at a single center were included in the study. All the predictors of the original risk score (age, diabetes mellitus, chronic obstructive pulmonary disease, congestive heart failure, chronic renal failure, old myocardial infarction, ejection fraction, intra-aortic balloon pump and peripheral arterial disease) were available for analysis. Results: Among the 2846 consecutive patients, a total of 1176 (41.3%) deaths were recorded during the 31,383 patient years of follow-up. The median EuroSCORE II was 2.35, and the median follow-up was 11.1 years. The risk score showed 72.7% overall discriminatory ability as measured by Harrell’s concordance statistic. It showed satisfactory calibration at 10, 15 and 20 years of follow-up. The risk score showed a time-varying nonlinear effect, and accordingly, adjusted long-term survival predictions were calculated. There were subgroups (scores < 50 points) with favorable 20-year survival rates ranging from 77% to 60%. Higher risk subgroups (scores > 90 points) showed poor 20-year survival rates ranging from 22% to 4%. Conclusions: The validated risk score represents a useful algorithm for the detection of patients who could benefit after CABG-BITA with respect to long-term survival. Although further multi-center studies are required worldwide to reveal the usefulness of this score in the clinical setting, its wide adoption may act as a motivation for cardiac surgeons resulting in higher numbers of CABG-BITA procedures.

## 1. Introduction

Nonrandomized clinical studies have shown the superiority of coronary artery bypass grafting (CABG) using bilateral internal thoracic artery (BITA) grafting (CABG-BITA) over conventional CABG with the use of a single internal thoracic artery plus vein grafts [1,2,3,4,5]. This finding has been attributed to the excellent long-term angiographic patency rates of the right internal thoracic artery, which appears to be equal to that of the left [6]. Although the randomized Arterial Revascularization Trial showed no significant difference in all-cause mortality or composite outcome at 10 years of follow-up in patients with CABG-BITA [7], it is possible that comparisons at longer follow-up time points (i.e., 15 or 20 years) may reveal its superiority. On the other hand, CABG-BITA represents a technically more demanding and time-consuming operation. The development of a risk stratification algorithm focusing on long-term outcomes in this subgroup of CABG patients may help clinicians, interventional cardiologists and cardiac surgeons in selecting patients who would benefit significantly from CABG-BITA.

Ziv-Baran et al. recently developed a simple nomogram for the prediction of long-term survival (up to 15 years) in patients who undergo isolated CABG-BITA [8]. Simplified additive scores are useful in the clinical setting because the predicted risk can be calculated easily and quickly, assisting in clinical decision-making for appropriate treatment intervention. In the present study, we performed external validation of this score in a large cohort of consecutive patients with isolated CABG-BITA. Furthermore, we evaluated the time-varying effect of the score in order to provide adjusted survival predictions up to 20 years after CABG-BITA.

## 2. Materials and Methods

### 2.1. Ethical Statement

The study was conducted in accordance with the Declaration of Helsinki, and it was presented to the Institutional Review Board, who approved the research protocol (11/3/2021, H-05-D-121). Waiver of the requirement for informed consent was granted because this is a retrospective observational study, and also anonymity was secured in the final database, which was used for statistical analyses.

### 2.2. Study Design

The current research represents an external validation study of a contemporary nomogram for the prediction of long-term survival after isolated CABG-BITA. This study is a type 4 prediction model study according to the Transparent Reporting of a multivariable prediction model for Individual Prognosis Or Diagnosis (TRIPOD) statement. We followed the recommendations for reporting the external validation of a predictive score without an update, as clearly outlined in the TRIPOD statement [9].

### 2.3. Patient Population and Data

This is a retrospective cohort study, and its population consists of consecutive patients who underwent isolated CABG-BITA between January 1992 and December 2008 at St. Luke’s Hospital Center, New York, NY, USA. This cardiac surgery database participates in the New York State Cardiac Surgery Reporting System and follows all audit processes to ensure completeness of data reporting, while all data were prospectively collected for clinical purposes. All the predictors of the original score were available for analysis, and there were no missing values in these variables. The Social Security Death Index was used to ascertain patients’ vital statuses through 28 February 2014. Social Security number alone has the best accuracy of any combination of other identifiers (first initial, last name, day, month, year of birth, etc.), with very high sensitivity and excellent specificity [10]. In this study, only Social Security numbers were used through our Java program for automatic obtaining of patient vital status and date of death from the web (https://www.genealogybank.com/explore/ssdi/all accessed on 21 March 2018), which returned the outcome and the dates in an anonymized way (not using names, and encoding Social Security numbers in a way not to be identifiable in the final dataset). Patients without Social Security numbers were followed until the time of discharge from the hospital. The endpoint of interest was all-cause mortality in the follow-up period. Preoperative risk stratification was performed according to the EuroSCORE II algorithm.

### 2.4. Statistical Analysis

Numerical variables were presented as median and interquartile range (IQR) and were compared with the Kruskal–Wallis test. Categorical variables were summarized by percentages and were compared with chi-square (linear by linear association) or Fisher’s exact test where appropriate. Harrell’s concordance (C) statistic was used to evaluate the overall discriminatory ability of the model. The area under the receiver operating characteristic curve (AUC) for time-dependent outcomes was used to calculate the discriminatory ability of the model at specific time points. The calibration of the model was evaluated graphically at specific time points with smoothed curves in order to avoid a statistical method that uses binning of continuous variables and, therefore, has lower precision than smooth estimates, which allow for interpolation. The time-varying, nonlinear effect of the score was explored using survival analysis with a generalized additive model, and it is represented as a three-dimensional surface and contour map with respective slices for fixed values of the score and fixed time points. Furthermore, predicted survival functions for the score with a time-varying nonlinear effect were calculated by splitting the database at failure events in order to fit a model with a time-varying nonlinear effect and draw the corresponding predicted survival curves. This approach required the analysis of 1,930,597 observations in a database with 1176 failure events. Finally, spline interaction analysis was conducted using natural splines by score in order to determine whether there is evidence for a score cut off for the benefit of male relative to female patients, as well as among various body mass index (BMI) groups.

All statistical analyses were performed with Stata (StataCorp. 2019. Stata Statistical Software: Release 16. College Station, TX: StataCorp LLC.) using the stroccurve, stcoxcal and scurve_tvc packages [11,12,13] and R (version 4.02, R Foundation for Statistical Computing, Vienna, Austria, 2020) including the survival (ver. 3.2–7), rms (ver. 6.0–1), mgcv (ver. 1.8–33), visreg (ver. 2.7.0), splines (ver.3.6.2) and pammtools (ver. 0.2.8) packages [14,15,16,17,18,19,20].

## 3. Results

Among the 2846 consecutive patients with isolated CABG-BITA, a total of 1176 (41.3%) deaths were recorded during the 31,383 patient years of follow-up. Patients without Social Security numbers (*n* = 89 or 3.1%) were followed until the time of discharge from the hospital. The median follow-up was 11.1 years (IQR = 6.6–16.2 years). Table 1 shows patient and disease characteristics in the quartiles of isolated CABG-BITA subgroups as determined by the predictive score, while the last column shows the points for each variable as developed in the original study.

As expected, all the variables of the original score showed statistically significant higher percentages from the low-risk to the high-risk quartiles. Female patients were also found in higher numbers in the higher-risk quartiles. There were more patients with normal BMI (18.5–25 kg/m^2^) in the high-risk quartile and fewer obese and morbidly obese patients in this quartile. The median EuroSCORE II ranged from 1.48 to 5.40 in the low-risk and high-risk quartiles, respectively. The median number of coronary arteries diseased was threein all quartiles, and the median number of distal anastomoses was four in all quartiles. There were a total of 54 (1.9%) in-hospital deaths, while the overall median EuroSCORE II was 2.35 (IQR = 1.44–4.15).

In comparison with the original development dataset, there were three predictors which showed a similar distribution in our dataset, including diabetes mellitus (33.7% vs. 32.7%), old myocardial infarction (39.2% vs. 34.9%) and mean age (65.2 vs. 64.5 years). There were four predictors which showed decreased distribution in our dataset, including chronic renal failure (7.2% vs. 2.9%), peripheral arterial disease (17.8% vs. 10.2%), congestive heart failure (20.8% vs. 12.6%) and intra-aortic balloon pump use (6.6% vs. 2.7%). Finally, there were two predictors with increased distribution, including chronic obstructive pulmonary disease (5.2% vs. 10.9%) and left ventricular ejection fraction ≤30% (7.4% vs. 18.2%).

**Figure 1:**
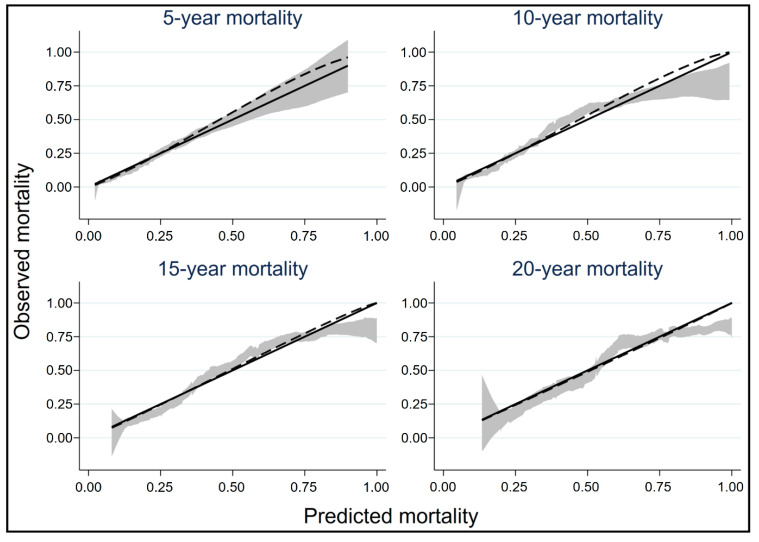
External calibration of the original score in the isolated CABG-BITA dataset at 5, 10, 15 and 20 years with smooth estimates with pointwise 95% confidence intervals plotted against predicted mortality probabilities. The solid line is the line of identity, denoting perfect calibration. Some miscalibration in the large is evident at 5 and 10 years with underprediction of mortality probabilities.

External validation of the original score was performed in our isolated CABG-BITA dataset. The overall discriminatory ability of the score as measured by Harrell’s C was 72.7% ± 0.7% (percentage ± standard error), slightly better than this in the study of Ziv-Baran et al. [8]. The discriminatory ability of the score at specific time points was evaluated by calculating the AUCs, and it was found to be very good (0.763, 0.772, 0.786 and 0.780 at 5, 10, 15 and 20 years of follow-up). The calibration of the score was also evaluated with a more robust method in a continuous manner (Figure 1), which showed that although the 95% confidence intervals of the predicted mortality probabilities include the line of identity (denoting perfect calibration) in most cases, there is some miscalibration in the large with underprediction of mortality at 5 and 10 years of follow-up. However, the lines are almost overlapping at 15 and 20 years of follow-up, indicating very good calibration of the model.

**Figure 2:**
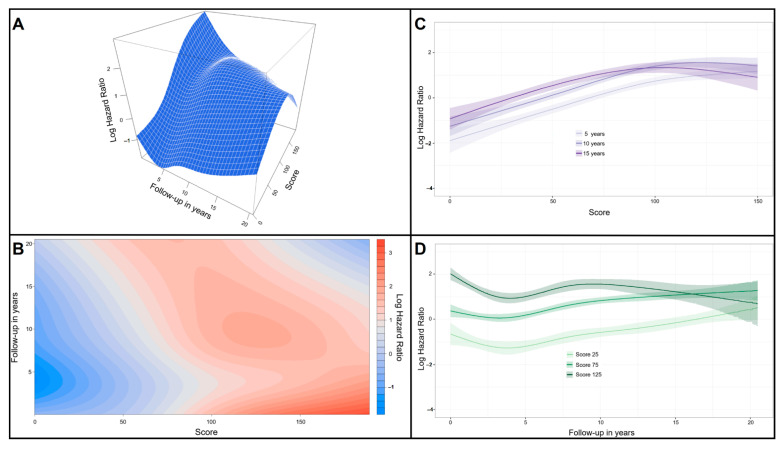
Three-dimensional surface plots, contour maps and representative slices for fixed values of the score and fixed time points showing the time-varying effect of the score. (**A**) Three-dimensional surface plot showing the relation between the score and follow-up time on the *y*- and *x*-axes, respectively. The z-axis shows the contribution of each combination on the log hazard ratio for all-cause mortality. (**B**) Contour map that uses a color gradient to visualize the effect of the combination on the log hazard ratio, where the darker shades of red denote an increase in the log hazard ratio. (**C**) Representative slices at fixed time points of 5, 10 and 15 years of the follow-up. The score and the log hazard ratio are shown in *x*- and *y*-axes, respectively. (**D**) Representative slices at fixed values of the score at 25, 75 and 125 points. The follow-up in years and the log hazard ratio are shown in *x*- and *y*-axes, respectively.

**Figure 3:**
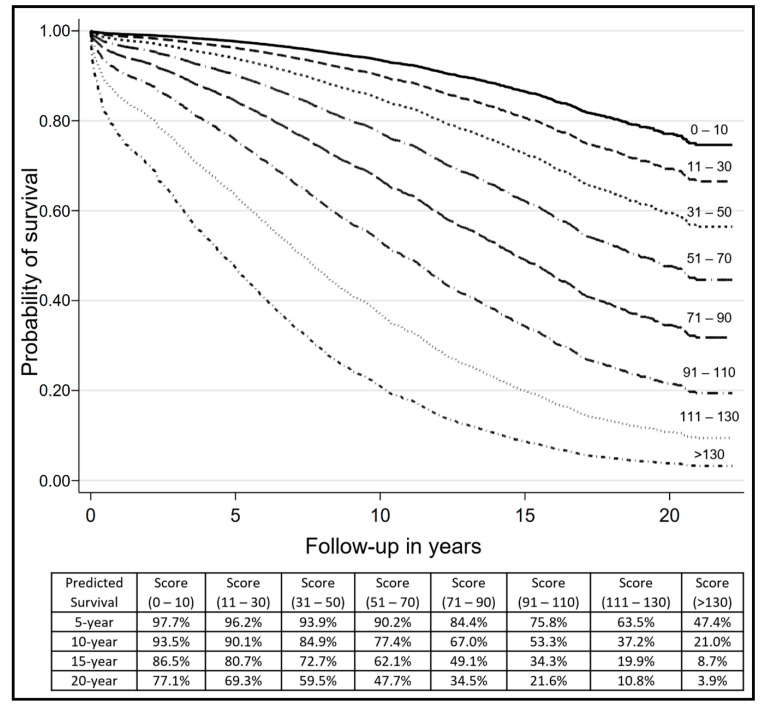
Prediction survival curves adjusted for the time-varying coefficient of the score in 8 subgroups according to the points total in the isolated CABG-BITA dataset (*n* = 2846). There were used cut off points at round numbers 0–10 (*n* = 233), 11–30 (*n* = 389), 31–50 (*n* = 414), 51–70 (*n* = 621), 71–90 (*n* = 502), 91–110 (*n* = 289), 111–130 (*n* = 259) and >130 (*n* = 139).

Figure 2 shows the pronounced time-varying effect of the score clearly in the three-dimensional surface plot. The contour map shows that the effect of the score fades over time. Representative slices at fixed time points of 5, 10 and 15 years of follow-up are shown to have an increasing effect on the score according to the points total. Representative slices at fixed values of the score at 25, 75 and 125 points are shown a decreased effect until the first 5 years for all scores, while lower scores (i.e., 25 and 75) are shown an increased effect thereafter in contrast to the higher score of 125 which continues to show a decreasing effect in the long-term follow-up. Then, predicted survival functions for the score were constructed after fitting a Cox model with a time-varying coefficient. Figure 3 shows the prediction survival curves adjusted for the time-varying coefficient of the score in eight subgroups according to the points total in the isolated CABG-BITA dataset, where it is shown that patients with scores up to 90 have a predicted survival of more than 50% at 15 years of follow-up, while patients with a score higher than 110 have a predicted survival less than 20% at the same time of follow-up. In addition, there were subgroups (scores < 50 points) with 20-year survival rates ranging from 77% to 60%.

**Figure 4:**
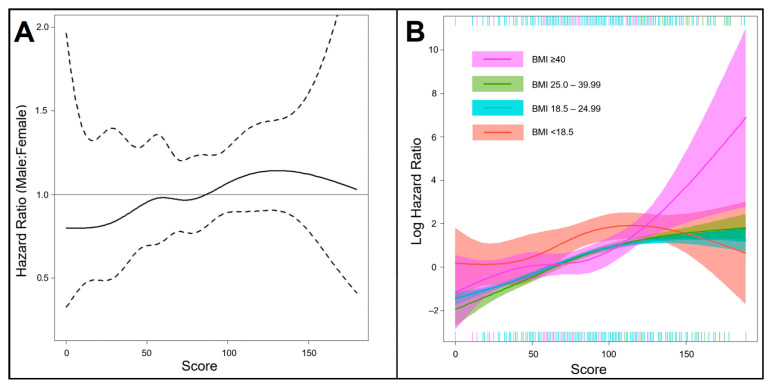
Spline interaction analysis. (**A**) Relationship of the hazard ratio in males relative to females with CABG-BITA across the score. The solid line is the hazard ratio, and the dotted lines are the 95% confidence intervals around the hazard ratio. A hazard ratio of more than 1 suggests increased all-cause mortality in males relative to females, as shown in this figure at higher than 85 points of the score. (**B**) Cross-sectional spline interaction analysis between the score (continuous variable) and the groups of BMI (categorical variable). The solid colored lines represent the log hazard ratios of the BMI categories, and the shadows are shown the corresponding 95% confidence intervals.

Female gender and BMI are well-known risk factors affecting the outcome of CABG; therefore, it assessed specifically the impact of the score on outcome in males relative to females, as well as among the four BMI groups. Figure 4A shows that CABG-BITA is associated with improved outcomes in females with a score higher than 85 compared to males. Morbid obesity (BMI ≥ 40 kg/m^2^) carries a very high risk of mortality in a score higher than 125, and underweight patients (BMI < 18.5 kg/m^2^) remain at higher risk for mortality at scores from 0 to 125 in comparison to all other BMI categories (Figure 4B).

## 4. Discussion

Contemporary published data from large observational studies and meta-analyses with follow-up exceeding the span of a decade consistently show the advantage of CABG-BITA in long-term survival [1,2,3,4]. On the other hand, this finding has not been confirmed by randomized studies as of yet [7], and in general, CABG-BITA has not been widely adopted by cardiac surgeons. Indeed, Zhu et al. showed that the use of BITA grafting among Medicare beneficiaries in the largest registry of CABG in the United States remained extremely low, counting for 2.1% in a series of 1,181,344 CABG patients from 1999 to 2010 [5]. However, after propensity matching in this dataset, BITA was also associated with improved long-term survival, which was independent of aortocoronary bypass grafts and diabetic status. It is obvious that a risk stratification algorithm for the subgroup of CABG-BITA may assist clinicians and surgeons in selecting those patients who would benefit from this type of surgery. Ziv-Baran et al. recently developed a simple nomogram for the prediction of long-term survival (up to 15 years) among patients with isolated CABG-BITA [8].

In the current study, we performed external validation of this score with respect to long-term (10, 15 and 20 years of follow-up) survival outcomes. First, the score was evaluated in the isolated CABG-BITA subgroup, where the model showed very good discriminatory ability at 10, 15 and 20 years of follow-up and satisfactory calibration. Second, the original score was presented with a time-varying nonlinear effect, and accordingly, we constructed adjusted survival predictions up to 20 years after CABG-BITA. The beneficial effect of CABG-BITA with respect to long-term all-cause mortality in stratified subgroups was clearly shown according to the score (Figure 3). Ziv-Baran et al. analyzed CABG-BITA cases from a single center, including consecutive patients from 1996 to 2011 [8]. Similarly, the current cohort of patients represents a single center’s dataset with consecutive isolated CABG-BITA cases from 1992 to 2008. The good performance of the original score in an external database is indicative of its generalizability. The different geographic origin of the external dataset represents another indication of a wide satisfactory performance of the original score. However, although CABG-BITA has not evolved much from a surgical point of view during the last decade, it remains to be seen from multi-center contemporary studies with long-term follow-up exceeding the 10-year mark, whether this score still performs well or whether it may need recalibration. In addition, female sex and BMI categories, which represent already known risk factors that affect long-term outcomes [21], were analyzed in an interactive manner with the score using natural splines.

The most recently published guidelines on myocardial revascularization recommend the use of BITA grafting in patients who do not have a high risk of deep sternal wound infection (Class IIa, Level of evidence B) [22]. More specifically, they clarify that BITA grafting should be considered depending on the patient’s life expectancy, risk of deep sternal wound infection, coronary anatomy, degree of target vessel stenosis, graft quality and surgical expertise. However, there are no specific criteria or algorithms for the detection of patients with favorable long-term survival, and therefore, it remains at the discrete preference of the surgeon to select patients suitable for CABG-BITA. As a general rule, BITA grafting is recommended in patients up to the age of 70 years old, and this is based on clinical evidence [23]. However, other studies have shown the beneficial effect of BITA grafting in long-term survival in high-risk subgroups of patients, including female patients, age above 75 years old, diabetes, urgent operation, impaired left ventricular ejection fraction, renal dysfunction, previous myocardial infarction and use of intra-aortic balloon pump [3,4]. The current study provides sufficient evidence that the evaluated score may help in closing this gap and, thus, in selecting patients with favorable long-term survival. For example, female patients with high BITA scores (>85) showed decreased hazard ratios for long-term mortality as compared with their male counterparts, and therefore, female patients with high predictive scores should not be excluded from BITA grafting.

By definition, this score consists of nine variables, and therefore, it evaluates the general condition of the patient with respect to long-term survival after CABG-BITA. This consideration highlights the importance of the points totals rather than evaluating every variable separately. We showed that a score higher than 110 is associated with less than 40% 10-year survival, and this criterion may be more robust than age per se. One of the major concerns that preclude cardiac surgeons from routine utilization of the BITA strategy is the risk of deep sternal wound infection [24]. However, in a published analysis of 24 studies with BITA grafting, it was shown that the skeletonization harvesting strategy was associated with significantly lower deep sternal wound infection rates [25]. This report was used among others in the recent guidelines on myocardial revascularization in order to recommend the skeletonized BITA dissection in patients with high risk for deep sternal wound infection complications (Class I, Level of evidence B) [22].

Kurlansky et al. and Saran et al. presented approximately 50% and 30% rates of CABG-BITA in their datasets, and they confirmed the superiority of BITA grafting [3,4]. In our dataset, we also have high adoption of this technique (59% in the entire isolated CABG dataset). It is possible that centers with high volumes of CABG-BITA may represent centers of excellence with many referrals for CABG-BITA and, therefore, should be adequately represented in randomized studies.

The current study has several limitations. First, this is a retrospective single-center investigation. Nevertheless, the information on preoperative risk factors was collected prospectively with the highly standardized audited methods of the New York State Cardiac Surgery Reporting System, which is one of the most robust systems worldwide. Second, the period of CABG-BITA surgeries analyzed in the present study was from 1992 to 2008. It is well perceived that there are refinements with respect to perioperative and immediate postoperative care over time, which may affect and improve outcomes. However, our analysis was focused on long-term survival outcomes, and therefore, early outcomes may not change significantly in long-term outcomes. Third, we did not examine intraoperative or postoperative predictors in order to maintain the preoperative usefulness of the score for long-term survival prediction purposes. Patient frailty was not evaluated in our study, and it may represent a selection bias from the surgeon’s point of view. Fourth, the surgical technique of BITA harvesting (pedicled vs. skeletonized) was not available, but in general, the most common technique used during that time was the pedicled one. Fifth, the configuration of the BITA grafting was not available in this database, and therefore, it was not possible to compare the durability of different BITA configurations. However, Magruder et al. showed that the configuration of BITA was not an independent predictor for long-term survival [26]. Sixth, the Social Security Death Index was used to ascertain patients’ vital statuses, and it analyzed all-cause mortality. Although the Social Security Death Index may be less accurate as compared to the National Death Index, its accuracy is high in elderly populations with cardiac surgery [27]. In addition, the reported survival rates at 5, 10 and 15 years of follow-up in patients with isolated CABG-BITA in the current study are almost identical as compared with those in the study by Ziv-Baran et al. (85.2%, 70.4% and 56.7% vs. 86.1%, 70.6% and 50.3%, respectively) [8]. Finally, all-cause mortality represents an acceptable long-term outcome for practical purposes in large-size datasets with a follow-up period extending beyond the 10-year time point. Authors should discuss the results and how they can be interpreted from the perspective of previous studies and of the working hypotheses. The findings and their implications should be discussed in the broadest context possible. Future research directions may also be highlighted.

## 5. Conclusions

The current study confirmed the satisfactory discriminatory ability and calibration of a simple additive score for the prediction of long-term survival in patients who undergo CABG-BITA. Furthermore, the time-varying nonlinear effect of the score was illustrated, and adjusted survival predictions up to 20 years were calculated. The evaluated score represents a useful algorithm for the detection of patients who could benefit significantly with respect to long-term survival after CABG-BITA. These predictions can be materialized in the preoperative period with only a few exceptions (i.e., emergent cases). The wide adoption of the score in the clinical setting may act as a motivation for cardiac surgeons to increase their numbers of CABG-BITA procedures. Further studies, preferably multi-center, are required in order to establish the usefulness of this score.

## Figures and Tables

**Table 1:** Patient characteristics in the quartiles of isolated coronary artery bypass with bilateral internal thoracic artery grafting.

Variable	Q1 Score 0–38 *n* = 810	Q2 Score 39–65 *n* = 615	Q3 Score 66–90 *n* = 734	Q4 Score > 91 *n* = 687	*p* Value	Points
Female sex, *n* (%)	144 (17.8)	141 (22.9)	216 (29.4)	244 (35.5)	<0.001	-
Age, years					<0.001	-
<55, *n* (%)	511 (63.1)	50 (8.1)	9 (1.2)	3 (0.4)	-	0
55–59, *n* (%)	144 (17.8)	220 (35.8)	42 (5.7)	15 (2.2)	-	29
60–64, *n* (%)	155 (19.1)	184 (29.9)	86 (11.7)	44 (6.4)	-	38
65–69, *n* (%)	0	161 (26.2)	250 (34.1)	109 (15.9)	-	58
70–74, *n* (%)	0	0	275 (37.5)	175 (25.5)	-	68
75–79, *n* (%)	0	0	72 (9.8)	189 (27.5)	-	90
≥80, *n* (%)	0	0	0	152 (22.1)	-	100
Diabetes mellitus, *n* (%)	106 (13.1)	226 (36.7)	248 (33.8)	352 (51.2)	<0.001	14
Chronic obstructive pulmonary disease, *n* (%)	31 (3.8)	42 (6.8)	67 (9.1)	171 (24.9)	<0.001	21
Congestive heart failure, *n* (%)	11 (1.4)	31 (5.0)	67 (9.1)	250 (36.4)	<0.001	14
Chronic renal failure	3 (0.4)	6 (1.0)	14 (1.9)	60 (8.7)	<0.001	19
Old myocardial infarction, *n* (%)	144 (17.8)	218 (35.4)	260 (35.4)	371 (54.0)	<0.001	11
Left ventricular ejection fraction ≤30%, *n* (%)	39 (4.8)	79 (12.8)	121 (16.5)	279 (40.6)	<0.001	18
Intra-aortic balloon pump, *n* (%)	6 (0.7)	15 (2.4)	16 (2.2)	41 (6.0)	<0.001	22
Peripheral arterial disease, *n* (%	11 (1.4)	37 (6.0)	48 (6.5)	193 (28.1)	<0.001	22
Body mass index, kg/m^2^					<0.001	-
<18.5, *n* (%)	5 (0.6)	7 (1.1)	5 (0.7)	13 (1.9)	-	-
18.5–24.99, *n* (%)	169 (20.9)	161 (26.2)	215 (29.3)	215 (31.3)	-	-
25.0–39.99, *n* (%)	618 (76.3)	427 (69.4)	496 (67.6)	447 (65.1)	-	-
≥40, *n* (%)	18 (2.2)	20 (3.3)	18 (2.4)	12 (1.7)	-	-
EuroSCORE II, median (IQR)	1.48 (1.04–2.35)	1.92 (1.33–2.88)	2.49 (1.61–3.87)	5.40 (3.44–9.06)	<0.001	-
Score, median (IQR)	18 (0–29)	54 (47–58)	79 (69–83)	112 (101–125)	<0.001	-
Number of coronary arteries diseased, median (IQR)	3 (2–3)	3 (3–3)	3 (3–3)	3 (3–3)	<0.001	-
Distal anastomoses, median (IQR)	4 (3–4)	4 (3–4)	4 (3–4)	4 (3–4)	0.035	-
In-hospital mortality, *n* (%)	5 (0.6)	7 (1.1)	9 (1.2)	33 (4.8)	<0.001	
Long-term mortality, *n* (%)	130 (16.0)	187 (30.4)	383 (52.2)	476 (69.3)	<0.001	-
Follow-up in years, median (IQR)	14.8 (9.4–18.5)	12.5 (7.9–16.9)	10.4 (6.6–14.4)	7.0 (3.0–11.2)	<0.001	-

For categorical variables, *n* (%) is presented. For continuous variables, median and interquartile range is presented. IQR = interquartile range; Q = quartile (Q1 = low-risk, Q2 = mild-risk, Q3 = medium-risk, Q4 = high-risk).

## Data Availability

The data underlying this article will be shared on reasonable request to the corresponding author.
